# Identification of COL1A1 associated with immune infiltration in brain lower grade glioma

**DOI:** 10.1371/journal.pone.0269533

**Published:** 2022-07-05

**Authors:** Junyu Ren, Junlong Da, Narisu Hu

**Affiliations:** 1 Oral Implant Center, Second Affiliated Hospital of Harbin Medical University, Harbin, Heilongjiang, China; 2 Institute of Hard Tissue Development and Regeneration, Second Affiliated Hospital of Harbin Medical University, Harbin, Heilongjiang, China; University of Nebraska Medical Center, UNITED STATES

## Abstract

Brain low grade gliomas (LGG) often give serious clinical symptoms due to the invasion towards nervous system, affecting the life quality of patients. Collagen type I alpha 1(COL1A1) is the main component of type I collagen. Although there are many reports about abnormal expression of COL1A1 in various tumors, specific role and clinical significance of COL1A1 in LGG have not yet been elucidated. In this work, Tumor Immune Estimation Resource database was used for detecting the expression level of COL1A1 in cancer and normal tissues, and aimed to explore the relationship between COL1A1 and tumor immune infiltration. We applied Kaplan-Meier to analyze the role of COL1A1 in clinical prognosis. Univariate survival rate and multivariate Cox analysis were used to compare clinical characteristics and survival rate. The relativity between the expression of COL1A1 and the tumor microenvironment was evaluated using ESTIMATE algorithm. Finally, the relationship between expression level of COL1A1 and gene marker sets of immune cell infiltration was investigated via TIMER. According to TCGA, COL1A1 overexpression was correlated with overall survival (OS), progression free interval (PFI) and disease specific survival (DSS) of multiple tumors, especially in LGG. Multivariate analysis showed that COL1A1 expression was an independent prognostic factor for LGG. The expression of COL1A1 was positively correlated with the infiltration of CD4 + T and CD8 + T cells, neutrophils, macrophages and dendritic cells in LGG. In addition, there was a strong correlation between expression of COL1A1 and different immune marker sets in LGG. The results suggest that COL1A1 is related with tumor immune infiltration of LGG.

## Introduction

Glioma is a primary tumor that originates from neuroglial stem or progenitor cells [1], which is common primary intracranial malignant tumor [2]. According to histological characteristics outlined by the World Health Organization (WHO), gliomas are classified into grades I to IV [3]. Grades I/II is classified as low-grade glioma (LGG), and grades III/IV is classified as high-grade glioma (HGG). Although LGG is less malignant than HGG, LGG inevitably develops into HGG eventually, and the long-term survival rate of LGG is still not satisfactory [4]. Immune mechanism plays a significant role in development of many tumors. Especially, LGGs, HGGs, and brain metastases lie on a spectrum of immunogenicity and T-cell infiltration [5]. Meanwhile, some immune cells, like T follicular helper (TFH) cells and M0 macrophages, had been selected as independent predictors for malignant transformation (MT) from LGG to HGG and formed an immune risk score [6]. In recent years, immunotherapy for the cancer has became as one of the most promising treatments. In these treatments for non-small cell lung cancer (NSCLC), immunotherapy using anti-programmed death-1 (PD-1) and its ligand PD-L1 antibody to target immune checkpoints is an effective method. [7]. This provides a new direction for the treatment of LGG. Therefore, there is an urgent need for new treatments that target the interaction between the tumor microenvironment (TME) and immune response.

Collagen is the major component of extracellular matrix (ECM) [8]. Type I collagen is widely distributed in structural organs such as bones, skin and teeth [9]. Type I collagen protein mainly consists of two collagen type I alpha 1 (COL1A1) and one collagen type I alpha 2 (COL1A2) chains [10, 11]. There is an evidence that the COL1A1 is involved in the carcinogenesis of several tissue types [12]. COL1A1 could promote the tumor progression in multiple types of cancer [13–15]. As an essential component of ECM, fibroblasts-secreted COL1A1 was proved to promote ovarian cancer metastasis via activating ITGB1/AKT signal pathway [16]. Meanwhile, COL1A1 is and involved in a variety of biological behaviors, such as cell proliferation, metastasis, invasion and angiogenesis [17, 18]. Bioinformatics analysis showed that there was differential expression of COL1A1 in gastric cancer [19]. However, it is unclear about the specific mechanism of COL1A1 in LGG and how it is related to tumor immunity.

Therefore, we employed databases of TCGA and Oncomine to comprehensively evaluate the correlation between the level of COL1A1 expression and prognosis of tumors. We also explored relationship between COL1A1 and tumor immune cell infiltration by relevant tumor data from TIMER. Our finding revealed the functional role of COL1A1 in LGG, and provided novel insights for LGG’s immunotherapy regimen.

## Materials and method

### 1. Differential expression of *COL1A1* by database analysis

We analyzed the differences in gene expression among the various type cancers from Cancer Genome Atlas (TCGA) project (https://portal.gdc.cancer.gov/repository) through Tumor Immune Estimation Resource (TIMER, https://cistrome.shinyapps.io/timer/) [20]. Through the Diff Exp module on the webpage, we check the differential expression of COL1A1 between the tumor and adjacent normal tissues. TIMER uses the Wilcoxon test by default to assess whether it is statistically significant. Considering the limited number of normal tissues sample in TCGA, we entered the Gene Expression Omnibus (GEO; https://www.ncbi.nlm.nih.gov/geo/query/acc.cgi?acc=GSE2223) and downloaded the GSE2223 data set, which contains 4 normal tissue samples and 26 LGG tumor samples. The information corresponding to the annotation samples was obtained through GPL1833, and the Wilcoxon test was applied to evaluate whether the expression of COL1A1 was different. Since the subtypes of LGG in the TCGA database is not comprehensive, we utilized the analysis module that comes with Chinese Glioma Genome Atlas (CGGA; http://www.cgga.org.cn/analyse/RNA-data.jsp) to analyze and visualize the mRNAseq_693 cohort in order to further explore the expression of COL1A1 in the subtypes [1–3].

### 2. Prognostic values of *COL1A1* analysis

Through the University of California Santa Cruz Xena browser (UCSC Xena, https://xenabrowser.net/datapages/) [21], we obtained the expression, clinical and survival data of 33 types of cancer. According to the data in the TCGA, Kaplan–Meier method was used to conduct univariate analysis of survival for overall survival (OS) by survival [22] and survminer [23] package in R software (version 3.6.3). Moreover, we applied the survival R package for Cox proportional hazards model for continuous variables, and the forestplot [24] R package for generating plots. Similarly, limma [25], survival, survminer and forestplot package were used to describe and analyze progression free interval (PFI), disease specific survival (DSS), disease free interval (DFI), and generate corresponding forest plots. We extracted clinical data of LGG on various tumors in the TCGA and CGGA database and deleted missing and incomplete data. Our research pays a special attention to the effect of COL1A1 on the cancer grade of LGG.

### 3. Tumor mutational burden and tumor microenvironment analysis

We downloaded the mutation data from the TCGA database and calculated the tumor mutational burden (TMB) score for each tumor, which was tested using Spearman’s rank correlation coefficient method. The tumor microenvironment consists of stromal cells, immune cells and cytokines, which has been shown to be closely related to the biological behavior of tumor cells [26]. The estimate [27] and limma packages in R software were also employed in evaluating the immune infiltration (ImmuneScore), overall stromal content (StromalScore) and the combined score (ESTIMATEScore) of the cancer samples in TCGA. Figures were created by R plotting system ggplot2 package [28].

### 4. Immune infiltration analysis

The Timer Web server is mainly used to systematically analyze the immune infiltration level of different tumors. The timer algorithm was applied to assess the abundance of 6 immune cells (CD4+T cells, CD8+T cells, B cells, neutrophils, macrophages, and dendritic cells). We conducted a series of analyses on the expression of COL1A1 in different tumors and infiltration abundance of immune cells in LGG via the gene module. Next, we collected 47 common immune checkpoint genes and estimated the relevance between COL1A1 expression and the immune checkpoint genes.

### 5. Statistical analysis

P-values of Kaplan–Meier survival, DSS, DFI and PFI analysis derived from a log-rank test. The cox regression model was performed for estimating the Hazard Ratio (HR) and 95% confidence interval (CI) values. The correlation of the expression levels of COL1A1 was analyzed by Spearman’s rank-order test correlation. P-values <0.05 were regarded as statistically significant.

## Results

### 1. *COL1A1* expression in different cancer

**Fig 1:**
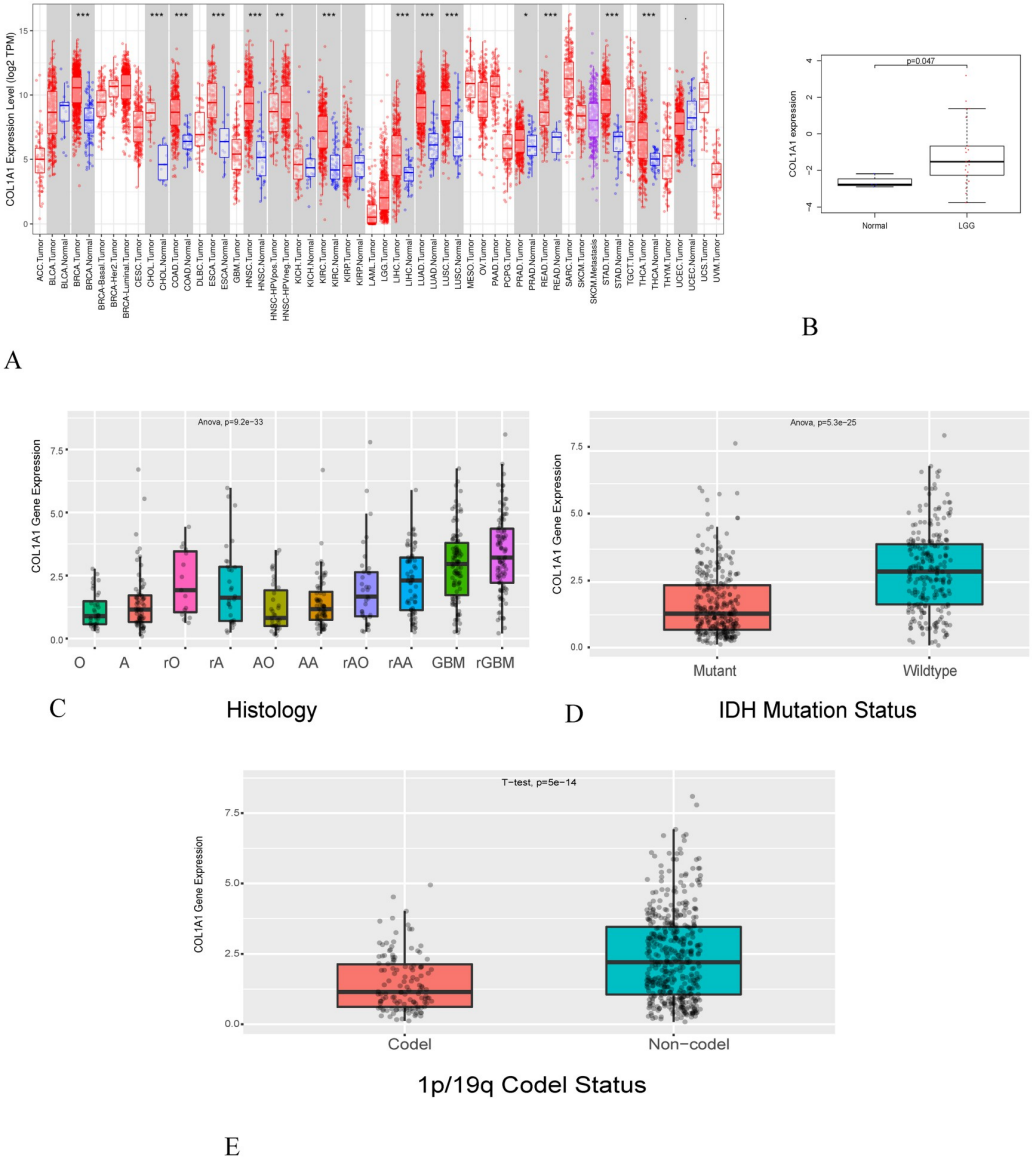
The expression level of COL1A1 in normal and tumor tissues. (A) In the TIMER database, the expression level of COL1A1 in different cancer tissues compared with normal tissues. (B) the expression level of COL1A1 in GEO. (C-E) The expression level of COL1A1 in different subtypes of glioma (A: Astrocytoma, O: Oligodendro, rO: recurrent Oligodendro, rA: recurrent Astrocytoma, AA: Anaplastic Astrocytoma, AO: Anaplastic Oligodendro, rAA: recurrent Anaplastic Astrocytoma, rAO: recurrent Anaplastic Oligodendro, GBM:Glioblastoma, rGBM: recurrent Glioblastoma.** P < 0.05,** P < 0.005, *** P < 0.001).

To estimate the expression level of genes in normal and tumor samples, we first conducted an analysis of multiple tumors using the TIMER database. The results demonstrated that the expression of COL1A1 was significantly increased in breast invasive carcinoma (BRCA), cholangiocarcinoma (CHOL), colon adenocarcinoma (COAD), esophageal carcinoma (ESCA), head and neck squamous cell carcinoma(HNSC), kidney renal clear cell carcinoma(KIRC), liver hepatocellular carcinoma (LIHC), lung adenocarcinoma (LUAD), lung squamous cell carcinoma (LUSC), prostate adenocarcinoma (PRAD), rectum adenocarcinoma(READ), stomach adenocarcinoma (STAD) and thyroid carcinoma (THCA) in Fig 1A as compared with normal tissues. We also applied GEO databases to estimate expression of gene in LGG. The results displayed that expression level of COL1A1 increased in LGG in contrast to normal tissues in Fig 1B. Considering that the number of normal glioma in TCGA is limited, we also referred to with CGGA database. We found that the expression of COL1A1 increased in LGG, including different histological subtypes and molecular classifications (Fig 1C–1E). It was worth noting that COL1A1 expressed highly in wildtype and Non-code, while Mutant and Code showed opposite trends (Fig 1D and 1E). In other words, the expression of COL1A1 was decreased in IDH-mutant and 1p/19q codeleted LGGs. Previous researchers found that IDH-mutant and 1p/19q codeleted LGGs have better prognosis [29], which further conformed our results. In summary, highly expressed COL1A1 played an important role on the patient’s prognosis.

### 2. Prognostic evaluation of *COL1A1* in tumors

**Fig 2:**
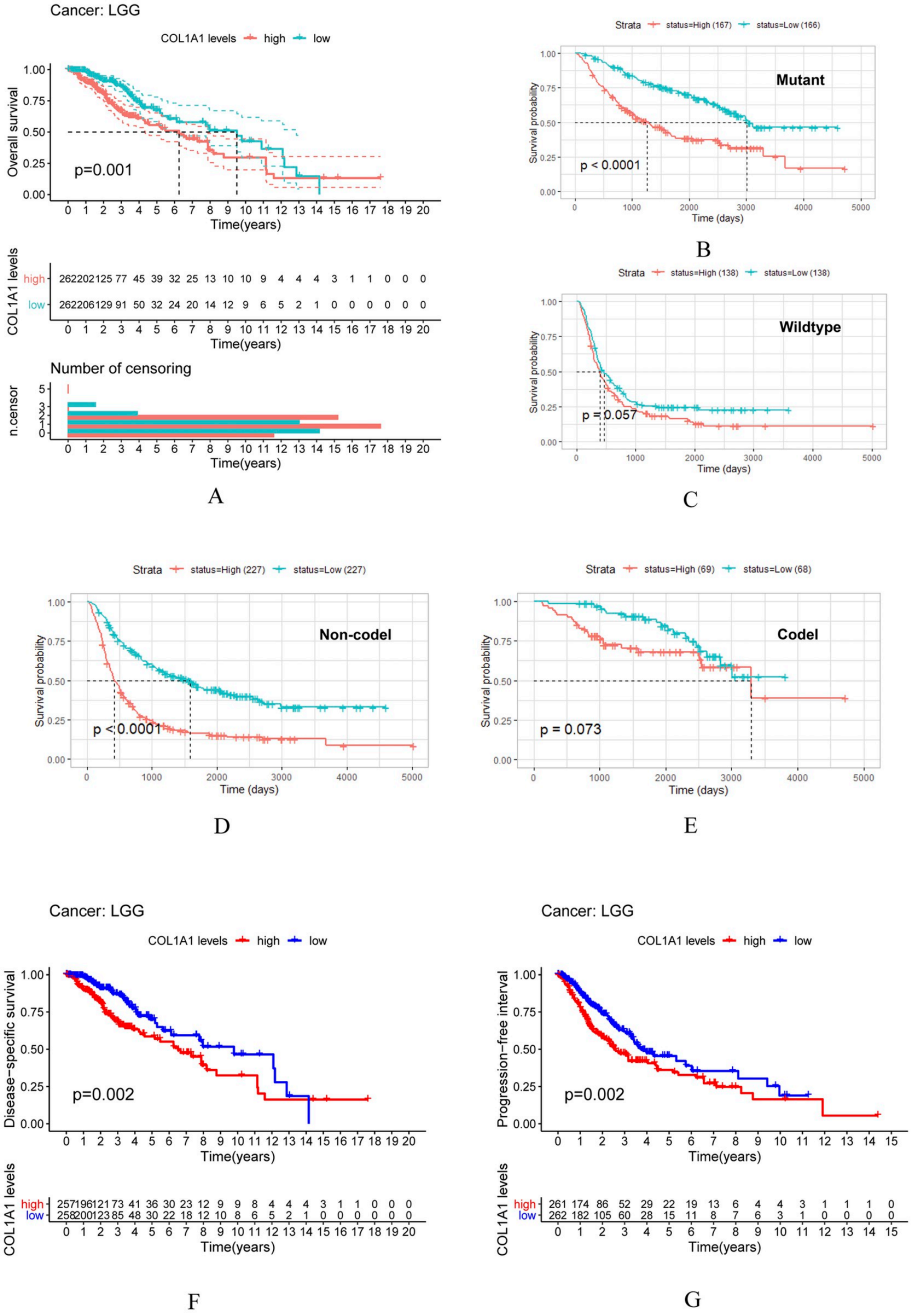
Overall survival curve of high and low expression of COL1A1 in glioma and it’s subclass. (A) Survival curves of OS by Kaplan-Meier respectively in LGG. (B-E) Survival curves of Survival probability in Mutant, Wildtype, Non-codel and Codel. (F-G) Survival curves of DSS and PFI in LGG. DSS, disease specific survival; PFI, progression free interval.

**Fig 3:**
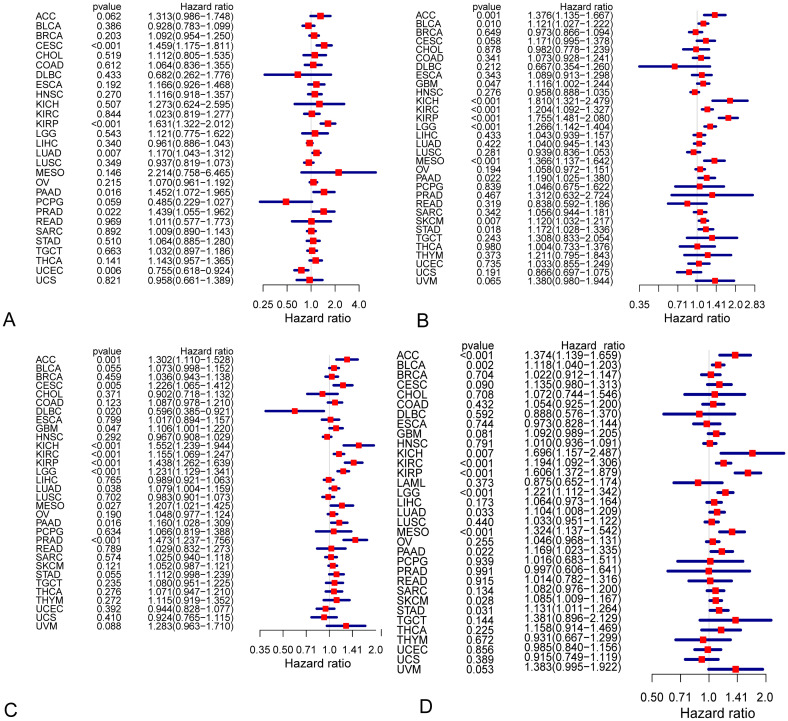
Survival curves and cox proportional hazards regression model of DFI, DSS, and PFI in different types of cancers. (A-C) Cox proportional hazards regression model of DFI, DSS, and PFI. (D) Cox proportional hazards model of OS in LGG for expression of COL1A1. DFI, disease free interval.

To investigate whether COL1A1 expression is correlated to the prognosis of tumor, we downloaded the clinical data of tumors from TCGA and CGGA. Figs 2 and 3 show the relationship between COL1A1 expression and the prognosis of different cancers. We found that the following four cancers showed an essential correlation between the prognosis of cancers and the expression of COL1A1, including LGG, KIRP, cutaneous melanoma (SKCM) and mesothelioma (MESO). The outcome indicates that highly expressed COL1A1 is significantly related to the poor prognosis of these cancers.

In order to evaluate the prognostic effect of the expression level of COL1A1 in different tumors, we show the overall survival curve of COL1A1 in LGG (Fig 2A) and in its subclass (Fig 2B–2E). It should be noted that overall survival curve of COL1A1 in Wildtype and Codel have a significant statistical significance (Fig 2B and 2D). However, we did not observe this phenomenon in Mutant and Codel (Fig 2C and 2E). Meanwhile the data source of Fig 2A is from TCGA dataset, Fig 2B and 2E are CGGA. Next, we used the other three outcome metrics provided by TCGA: DSS, PFI and DFI. These results clearly indicate that the expression of COL1A1 has an effect on the prognosis of LGG (Fig 2F and 2G). Although, we did not ascertain any significant relationship between the expression of COL1A1 and DFI in LGG patients (Fig 3A), it was worth noting that high COL1A1 expression relates to DSS and PFI in multiple cancers including LGG (Fig 3B and 3C). Meanwhile, R software was used to detect the prognostic value of COL1A1 by cox proportional hazards model (Fig 3D). Unanimously, the higher expression level of COL1A1 was shown to be related to its poor prognosis in LGG (OS HR = 1.221, 95% CI = 1.112 to 1.342, P = 3.00E-05).

**Fig 4:**
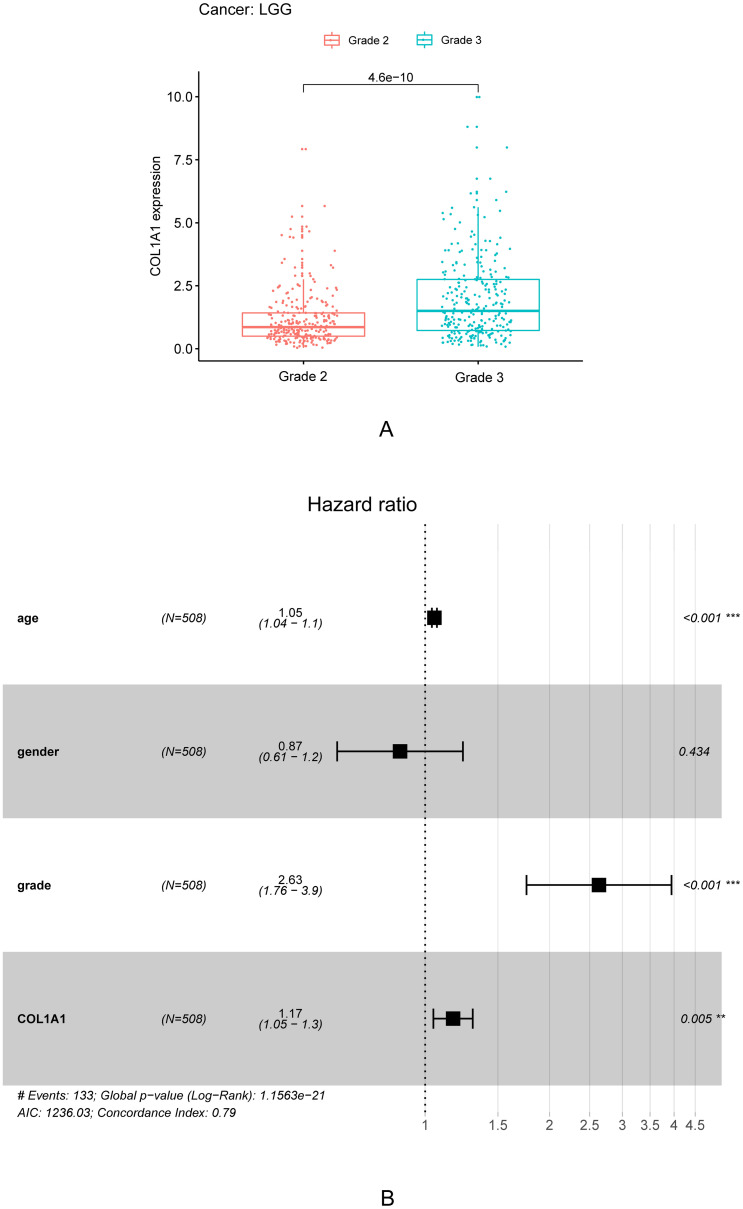
The expression of COL1A1 in LGG grade and the forest plot of LGG. A. Differential expression of COL1A1 in different cancer grade of LGG. Based on data from 258 grade2(G2) patients and 270 grade3(G3) patients in TCGA. B. Multivariate Cox analysis of COL1A1 expression and clinical pathological factors. As age, tumor grade and COL1A1 expression are independent prognostic factors (* P <0.05; ** P < 0.005;*** P < 0.001).

In addition, we also collected and compiled clinical data. As shown in Table 1, univariate correlation analysis using Cox regression showed that the grade was significantly correlated with overall survival. Age, tumor grade, and COL1A1 expression were independent prognostic factors in multivariate analysis (Fig 4B). The expression of COL1A1 showed a significant correlation with tumor grade through Table 1, Fig 4. With the increase of tumor grade, the expression of COL1A1 increased accordingly (Fig 4A).

**Table 1:** Univariate (a) and multivariate analysis (b) of the correlation of COL1A1 expression with OS among LGG patients.

	Hazard Ratio(HR)	95%CI	pvalue
a			
age	1.056	1.042–1.070	0.000
gender	0.857	0.607–1.211	0.381
grade	3.496	2.389–5.115	0.000
COL1A1	1.264	1.144–1.398	0.000
b			
grade	2.633	1.758–3.944	0.000
COL1A1	1.168	1.047–1.304	0.005

### 3. TMB and TME analysis

**Fig 5:**
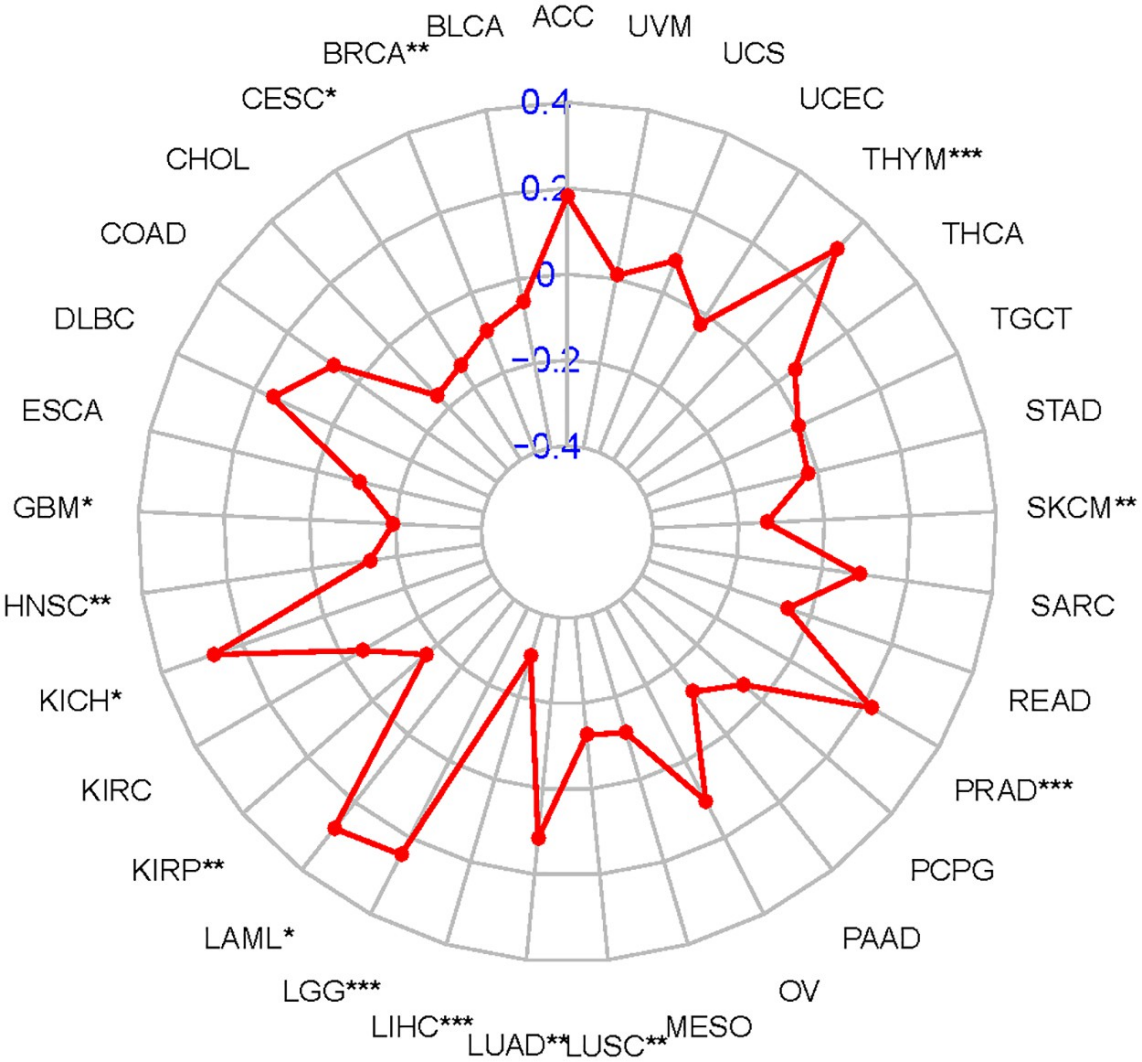
Radar graph for relationship between the expression of COL1A1 and TMB of each tumor sample. Correlation between Tumor mutational burden and LGG, THYM, PRAD, LUAD, LAML and KICH(* P <0.05; ** P < 0.005;*** P < 0.001).

TMB is an independent biomarker that can be used to predict the efficacy of immunotherapy found in various tumor in recent years. Those with high TMB expression have greater clinical benefit from immune checkpoint inhibitor treatment [30]. Here, we separately counted the TMB of each tumor sample, and analyzed the relationship between gene expression and TMB as follows, using Spearman rank correlation coefficient (Fig 5). We can see that there are six tumors that are positively correlated with TMB from the analysis, including LGG, thymoma (THYM), PRAD, LUAD, acute myeloid leukemia (LAML) and kidney chromophobe (KICH). These results indicate that the treatment plan of immune checkpoint inhibitor may play a significant role in these tumors.

**Fig 6:**
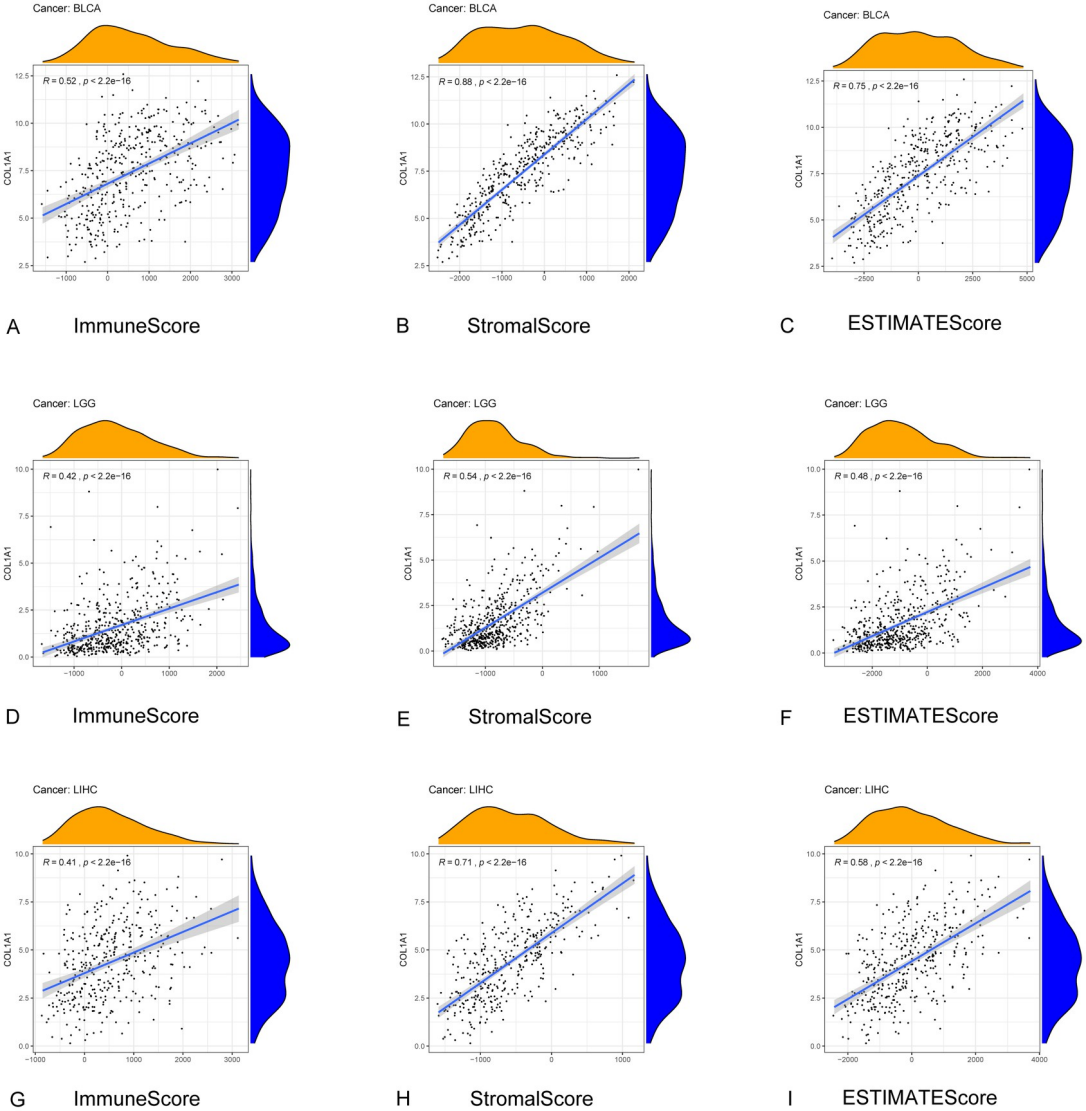
Correlation between COL1A1 expression and TME in diffrent tumer. (A-C). Relationship between COL1A1 expression and Stromal Score, Immune Score and ESTIMATE Score in BLCA. (D-F). Correlation between COL1A1 expression and Stromal Score, Immune Score and ESTIMATE Score in LGG. (G-I). Relationship between COL1A1 expression and TME in LIHC.

TME is the cellular environment of tumor. It’s composed of immune cells, inflammatory mediators, endothelial cells, extracellular matrix (ECM) and mesenchymal cells [31]. In TME, stromal and immune cells are two major non-tumor components, which are considered to be of great significance for the diagnosis and prognostic evaluation of cancers. We calculated the correlations between gene expression levels and ImmuneScore, StromalScore respectively to predict the infiltration of stromal and immune cells by the ESTIMATE algorithm among 33 cancers (Table 2). We selected several representative results for visualization (Fig 6). The results reveal that the expression of COL1A1 has a strong correlation with the TME of various cancers.

**Table 2:** Correlation analysis of COL1A1 expression level and TME score.

CancerType	StromalScore P value	cor	ImmuneScore P value	cor	ESTIMATEScore P value	cor
ACC	**	0.389	0.768	0.034	0.077	0.200
BLCA	***	0.883	***	0.521	***	0.748
BRCA	***	0.770	***	0.214	***	0.495
CESC	***	0.695	0.886	-0.008	***	0.328
CHOL	*	0.478	0.460	0.127	0.084	0.292
COAD	***	0.796	***	0.475	***	0.685
DLBC	***	0.674	0.088	-0.249	0.068	0.266
ESCA	***	0.742	*	0.205	***	0.521
GBM	***	0.636	***	0.353	***	0.485
HNSC	***	0.822	*	0.137	***	0.500
KICH	***	0.560	*	0.344	**	0.454
KIRC	***	0.711	***	0.214	***	0.467
KIRP	***	0.562	***	0.243	***	0.388
LAML	*	0.247	0.973	-0.003	0.197	0.106
LGG	***	0.544	***	0.421	***	0.480
LIHC	***	0.712	***	0.406	***	0.578
LUAD	***	0.553	**	0.169	***	0.380
LUSC	***	0.764	***	0.355	***	0.568
MESO	***	0.635	0.982	-0.002	*	0.302
OV	***	0.835	***	0.286	***	0.592
PAAD	***	0.659	***	0.354	***	0.521
PCPG	***	0.631	***	0.441	***	0.561
PRAD	***	0.752	***	0.390	***	0.604
READ	***	0.836	***	0.404	***	0.687
SARC	***	0.495	0.056	0.118	***	0.275
SKCM	***	0.689	***	0.203	***	0.415
STAD	***	0.630	***	0.215	***	0.451
TGCT	***	0.738	***	-0.359	0.440	0.062
THCA	***	0.759	***	0.587	***	0.697
THYM	***	0.775	0.551	-0.055	***	0.416
UCEC	***	0.624	*	0.123	***	0.357
UCS	***	0.788	0.030	0.292	***	0.557
UVM	***	0.666	**	0.402	***	0.506

* P <0.05;

** P < 0.005;

*** P < 0.001.

### 4. *COL1A1* expression correlated with immune cell infiltration and immune cell markers in LGG

In cancer patients, tumor-infiltrating lymphocytes are major indicators that independently predict the overall survival rate and sentinel lymph node status [32, 33]. Thus, we further explored if the COL1A1 expression is correlated with the degree of immune cell infiltration in different types of tumor by TIMER database.

**Fig 7:**
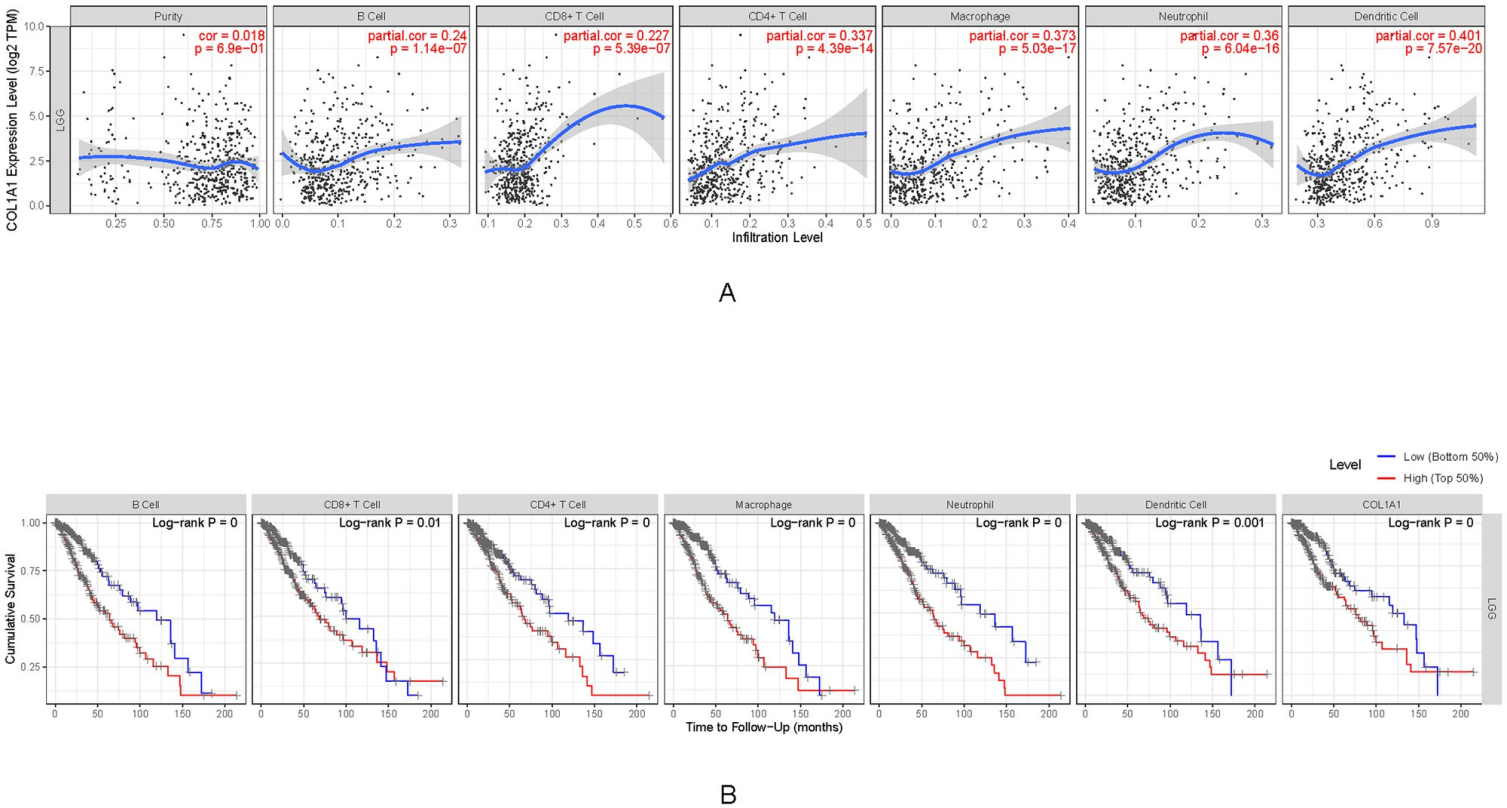
Immune infiltration level and survival time in LGG. A. Correlation of COL1A1 expression with infiltrating levels of B cell, CD4+ T cells, CD8+ T cells, Macrophages, Neutrophils and DCs in LGG. B. Cumulative survival of COL1A1 expression related to B cell, T cells, Macrophages, Neutrophils and DCs in LGG.

In the LGG, the expression of COL1A1 was notably associated with the infiltration levels of B cell (R = 0.24, P = 1.14e−07), CD4+T cells (R = 0.337, P = 4.39e−14), CD8+ T cells (R = 0.227, P = 5.39e−07), macrophages (R = 0.373, P = 5.03e−17), dendritic cells (R = 0.401, P = 7.57e−20) and neutrophils (R = 0.36,P = 6.04e−16) (Fig 7A). To further clarify the correlation between COL1A1 and immune cell infiltration in LGG, we generated the Kaplan-Meier diagram through the TIMER database. We found that B cell (P = 4.25E-05), CD8+ T cells(P = 0.0095), CD4+T cells(P = 0.0005), macrophages(P = 9.20E-06), neutrophils(P = 5.83E-06), dendritic cells infiltration (P = 0.0006), and COL1A1 expression (P = 0.0004) were significantly correlated with the prognosis of LGG(Fig 7B). It indicates that COL1A1 clearly plays an important role in modulating immune cell infiltration in LGG, especially in macrophages and DCs infiltration.

Next, in order to explore the relationship between a variety of immune infiltrating cells and COL1A1 in TIMER, we pay attention to the correlation between COL1A1 and the gene sets of immune cell marker in LGG and MESO (control group). We analyzed the correlation between different immune cell marker genes and COL1A1, such as CD8+ T cells, T cells (general), B cells, monocytes, TAMs, M1 and M2 macrophages, neutrophils, NK cells, DCs, Th1 cells, Th2 cells, Tfh cells, Th17 cells, Tregs and exhausted T cells. According to the results adjusted for purity, the expression level of COL1A1 in LGG was significantly correlated with most of the immune cell marker sets (Table 3). However, expression of COL1A1 was significantly correlated with only 9 immune cell marker sets in MESO. Specifically, the results show that COL1A1 is significant correlated with CD8A, CD8B of CD8+ T cell markers, CD3D, CD3E, CD2 of T cell markers, CD19, CD79A of B cell markers, CD86, CD115 of monocyte markers, CCL2, CD68, IL10 of TAM markers, IRF5, COX2 of M1 macrophages markers, CD163, VSIG4, MS4A4A of M2 macrophage markers, CD11b, CCR7 of neutrophils markers, KIR2DL1, KIR2DL3, KIR2DL4, KIR3DL1, KIR3DL2, KIR2DS4 of natural killer cell markers, HLA-DPB1, HLA-DQB1, HLA-DRA, HLA-DPA1, BDCA-1, BDCA-4, CD11c of DCs markers, T-bet, STAT1, IFN-γ of Th1 markers, GATA3, STAT6, STAT5A of Th2 markers, STAT3 of Th17 markers, CCR8, TGFβ of Treg markers and PD-1, CTLA4, LAG3, TIM-3, GZMB of T cell exhaustion markers in LGG.

**Table 3:** Correlation analysis of COL1A1 expression level and immune cell markers in TIMER.

Description	Gene markers	LGG				MESO			
		None		Purity		None		Purity	
		Cor	P	Cor	P	Cor	P	Cor	P
CD8+ T cell	CD8A	0.230	***	0.253	***	0.024	0.825	-0.101	0.357
	CD8B	0.194	***	0.205	***	0.117	0.281	0.020	0.855
T cell (general)	CD3D	0.491	***	0.511	***	0.061	0.573	-0.064	0.561
	CD3E	0.518	***	0.531	***	0.081	0.457	-0.047	0.671
	CD2	0.544	***	0.554	***	0.031	0.775	-0.090	0.412
B cell	CD19	0.372	***	0.352	***	0.015	0.893	-0.112	0.309
	CD79A	0.218	***	0.221	***	-0.122	0.259	-0.239	0.027
Monocyte	CD86	0.285	***	0.301	***	0.061	0.574	-0.020	0.856
	CD115 (CSF1R)	0.172	***	0.184	***	0.190	0.078	0.130	0.237
TAM	CCL2	0.358	***	0.353	***	-0.147	0.175	-0.187	0.086
	CD68	0.401	***	0.417	***	0.043	0.696	-0.012	0.916
	IL10	0.358	***	0.361	***	0.139	0.199	0.078	0.477
M1 Macrophage	INOS (NOS2)	-0.042	0.345	-0.029	0.533	0.459	***	0.453	***
	IRF5	0.287	***	0.324	***	-0.189	0.080	-0.234	0.031
	COX2(PTGS2)	0.148	**	0.143	*	0.225	0.037	0.253	0.019
M2 Macrophage	CD163	0.480	***	0.463	***	0.138	0.203	0.058	0.600
	VSIG4	0.213	***	0.212	***	0.142	0.190	0.100	0.364
	MS4A4A	0.422	***	0.424	***	0.174	0.107	0.090	0.415
Neutrophils	CD66b (CEACAM8)	-0.025	0.571	-0.038	0.407	0.099	0.360	0.127	0.246
	CD11b (ITGAM)	0.256	***	0.281	***	0.193	0.074	0.154	0.160
	CCR7	0.391	***	0.391	***	0.045	0.681	-0.092	0.403
Natural killer cell	KIR2DL1	0.164	**	0.168	**	-0.213	0.048	-0.286	*
	KIR2DL3	0.266	***	0.266	***	-0.206	0.056	-0.272	0.012
	KIR2DL4	0.278	***	0.272	***	-0.289	**	-0.385	**
	KIR3DL1	0.146	**	0.148	*	-0.184	0.088	-0.284	*
	KIR3DL2	0.190	***	0.209	***	-0.080	0.462	-0.181	0.098
	KIR3DL3	0.032	0.470	0.033	0.476	-0.329	**	-0.400	**
	KIR2DS4	0.209	***	0.226	***	-0.142	0.190	-0.190	0.082
Dendritic cell	HLA-DPB1	0.499	***	0.506	***	0.164	0.130	0.087	0.426
	HLA-DQB1	0.428	***	0.425	***	0.129	0.233	0.078	0.477
	HLA-DRA	0.498	***	0.503	***	0.149	0.169	0.070	0.526
	HLA-DPA1	0.506	***	0.513	***	0.148	0.171	0.063	0.565
	BDCA-1(CD1C)	0.379	***	0.357	***	0.181	0.093	0.130	0.236
	BDCA-4(NRP1)	0.521	***	0.519	***	0.567	***	0.546	***
	CD11c (ITGAX)	0.232	***	0.256	***	0.062	0.566	-0.005	0.967
Th1	T-bet (TBX21)	0.448	***	0.438	***	-0.103	0.342	-0.269	0.013
	STAT4	-0.066	0.134	-0.057	0.214	0.036	0.739	-0.112	0.307
	STAT1	0.429	***	0.427	***	-0.138	0.203	-0.218	0.046
	IFN-γ (IFNG)	0.214	***	0.226	***	0.076	0.484	-0.023	0.837
	TNF-α (TNF)	0.061	0.169	0.052	0.252	0.026	0.815	-0.105	0.338
Th2	GATA3	0.407	***	0.421	***	0.243	0.023	0.208	0.056
	STAT6	0.241	***	0.304	***	-0.134	0.214	-0.113	0.302
	STAT5A	0.299	***	0.311	***	-0.168	0.121	-0.209	0.054
	IL13	-0.066	0.133	-0.051	0.266	0.130	0.230	0.137	0.210
Tfh	BCL6	-0.042	0.344	-0.065	0.155	0.177	0.101	0.209	0.055
	IL21	0.091	0.039	0.080	0.080	0.108	0.317	0.113	0.304
Th17	STAT3	0.469	***	0.456	***	0.107	0.325	0.072	0.515
	IL17A	0.045	0.304	0.029	0.523	0.084	0.441	0.123	0.262
Treg	FOXP3	0.045	0.307	0.069	0.130	0.224	0.037	0.167	0.127
	CCR8	0.162	**	0.173	**	0.227	0.034	0.163	0.135
	STAT5B	0.019	0.669	0.012	0.797	0.210	0.050	0.205	0.060
	TGFβ (TGFB1)	0.340	***	0.353	***	0.488	***	0.474	***
T cell exhaustion	PD-1 (PDCD1)	0.430	***	0.428	***	0.031	0.777	-0.036	0.741
	CTLA4	0.322	***	0.309	***	0.117	0.280	0.022	0.844
	LAG3	0.300	***	0.294	***	-0.285	**	-0.377	**
	TIM-3 (HAVCR2)	0.312	***	0.336	***	0.058	0.593	-0.025	0.818
	GZMB	0.425	***	0.425	***	-0.225	**	-0.357	**

* P <0.05;

** P < 0.005;

*** P < 0.001.

The expression of COL1A1 is particularly related to markers of the most TAM, monocyte, M1 and M2 macrophage in LGG. It further suggests that COL1A1 may be involved in the polarization process of macrophages. On the basis of these results, we find that the marker gene sets of DCs are significantly associated with COL1A1, suggesting that high expression level of COL1A1 is correlated to the increase of DCs infiltration in LGG, but this requires further research to support this conclusion. We also observed that COL1A1 is significantly related to the T cell exhaustion marker gene sets, indicating that COL1A1 may play a crucial part in immune escape of LGG.

### 5. *COL1A1* expression correlated with Immune checkpoint gene in LGG

**Fig 8:**
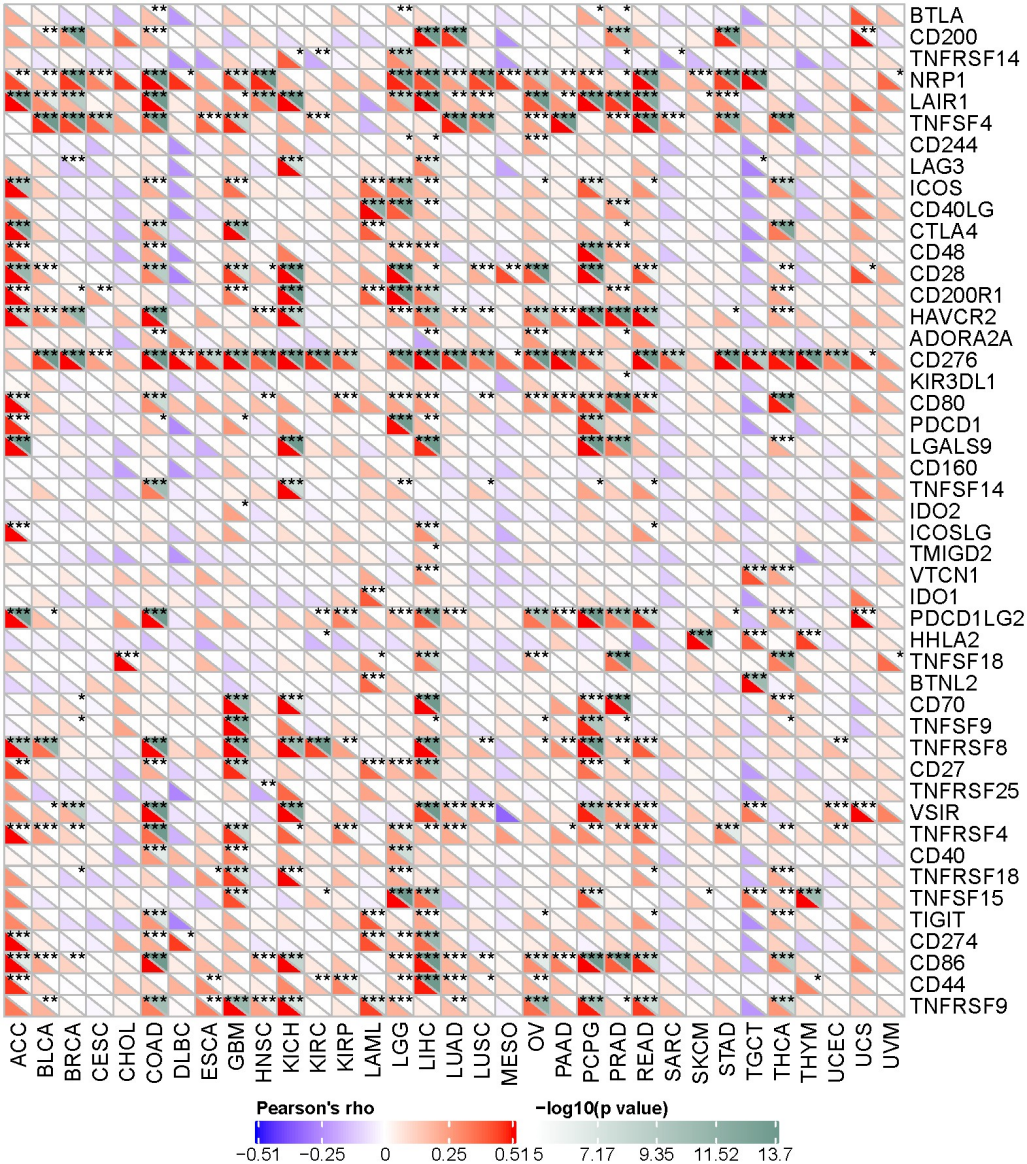
Correlation of the expression between COL1A1 and immune checkpoint genes in different tumors. Based on Pearson correlation coefficients, COL1A1 has a significant correlation with most selected immune checkpoint genes in LGG(* P <0.05; ** P < 0.005;*** P < 0.001).

Immune checkpoint pathway used by cancers inhibit tumor-reactive T cells considering as a mechanism of immune resistance, and it may induce immune suppression and immune escape [34]. Immune checkpoint inhibitors (ICIs) are one of the most promising methods for treating cancer [35]. To identify potential therapeutic targets of those cancers and immune escape, we have collected more than forty representative immune checkpoint genes, including BTLA, CD200, TNFRSF14, NRP1, LAIR1 and so on, and we evaluated the relationship between expression of COL1A1 and immune checkpoint. We extracted these immune checkpoint genes separately and calculated the coefficient correlation with expression of our target gene. The most of the immune checkpoint genes were observed to be positively co-expressed with COL1A1 of 33 tumors. We found that the immunological target showing remarkably significant correlation is CD276 among 27 tumors. In LGG, COL1A1 has a significant correlation and statistical significance with most selected immune checkpoint genes (Fig 8). Among them, CD28(R = 0.82,P = 6.37E-128),NRP1(R = 0.49, P = 5.92E-31), PDCD1(R = 0.51, P = 8.33E-34) and TNFSF15(R = 0.75, P = 1.55E-92) have a strong correlation with COL1A1 expression. Accordingly, the results further confirmed the specific correlation between COL1A1 and immune infiltrating in LGG, indicating that COL1A1 has a profound effect on immune escape of TMB.

## Discussion

COL1A1, located in chromosome 17, region 17q21–22, and presenting 51 exons, encodes the pro-α1 chain of type I collagen, which is consisting of a triple helix composed of one α2 chain and two α1 chains [36]. A fibrillar collagen C‑terminal domain (COLF) a collagen triple‑helix repeat and A von Willebrand factor type C (vWFC) domain are three conservative domains of COL1A1 [37]. Previous studies have shown that the expression of COL1A1 is correlated to a variety of tumor types, and COL1A1 is highly expressed in tumor tissues and cells [38–43]. Based on the integrated transcriptome analysis, six crosstalk collagen genes (including COL1A1) may be related to the progress and prognosis of LGG (4). Functional enrichment analysis showed that over-expressed COL1A1 may participate in the progression and poor prognosis of LGG through the ECM-receptor interaction pathway. COL1A1 regulates the expression of three key genes of Rac1-GTP, p-JNK and RhoA-GTP in WNT/PCP signaling pathway. The Wnt/PCP signaling pathway mainly activates JNK through small G protein to regulate cytoskeletal rearrangement, and transmits signals from Frizzled and ROR2/RYK co-receptors on the cell surface to the nucleus through Rho GTPases and JNK, which is essential for cell migration indispensable process [44]. Among these key proteins, JNK is central to promoting tumor invasion and metastasis [45]. Therefore, COL1A1 may affect tumor development through WNT/PCP signaling pathway. However, there are few reports on the function and mechanism of COL1A1 in LGG. Recent studies have shown that collagen density is related to the infiltration of T cells, thereby affecting tumor development [7]. In this research, we are aimed at exploring the expression level of COL1A1 and prognosis in LGG, and analyze the related mechanism of COL1A1 in tumor immune infiltration.

In this report, differences in expression levels of COL1A1 are related to the prognosis of different tumors, which is consistent with previous literature results. We find that COL1A1 has higher expression levels in multiple cancer tissues and the highly expressed COL1A1 is closely correlated to the poor prognosis of LGG. In addition, our results indicate that COL1A1 expression is interrelated with numerous immune marker sets and immune infiltration levels of LGG. Thus, our research suggests that COL1A1 may have a potential role in tumor immunology and provide a theoretical basis for being a biomarker of LGG. To further investigate the potential mechanism of COL1A1 expression in LGG and its clinical correlation, we got the dataset from TCGA database. The expression of COL1A1 was associated with the grade of this tumor by the relative R package analysis, and the study indicated that expression of COL1A1 was a critical independent prognostic factor through multivariate analysis in patients of low-grade glioma.

In this research, we used TCGA independent data sets and GEO database to assess the expression of COL1A1 and the prognosis in kinds of tumor, showing that the expression of COL1A1 of multiple cancers is significantly different between tumors and normal tissues. Then, we analyzed 33 cancers in the TCGA database and observed that expression of COL1A1 was higher in CHOL, COAD, LIHC, STAD, THCA, ESCA, LUSC, HNSC, BRCA, KIRC, LUAD, PRAD and READ compared with adjacent normal tissues. Due to the differences in collection methods of different database and potential biological characteristics, there are certain differences in the expression of COL1A1. However, we observed a correlation between increased expression of COL1A1 and poor prognosis of LGG. In mutant group and non codel group, the high expression level of COL1A1 showed significant low survival rate (P < 0.05), but there was no significant difference in wildtype group and codel group(P>0.05). Through the clinical prognosis data sets in the TCGA database, we found that the HR value of COL1A1 in OS, DSS, PFI revealed that the high expression level of COL1A1 is a high risk factor of the prognosis in LGG. Therefore, these results suggest that COL1A1 could be a valuable factor for the prognosis of LGG.

Another aspect of this study was to estimate the relevance between the COL1A1 and tumor immune infiltration, especially in LGG. We noticed that the degree of macrophage, CD8+, CD4+, DCs and neutrophil infiltration was positively correlated with expression level of COL1A1 via TIMER. Further research confirmed that these immune cells are closely related to the prognosis of LGG. Moreover, we can conclude from the relationship between the expression level of COL1A1 and certain markers of immune cell that COL1A1 plays a critical role in regulating tumor immunology of LGG. In the study of gene markers of macrophages, we found that the expression of COL1A1 is weakly correlated with M1 macrophage gene markers (IRF5, COX2), but it is more moderately correlated with M2 macrophage gene markers(CD163, MS4A4A), which suggests that COL1A1 may be potential regulatory role in polarization of tumor-associated macrophages(TAM). Additionally, our further results show that the increased expression of COL1A1 is positively correlated with the T cell exhaustion markers (PD-1, LAG3, CTLA4, TIM-3 and GZMB) in LGG, thus the T cell exhaustion is associated with the potential of COL1A1 expression. The PD-1L/PD-1 immune checkpoint is regarded as a target for immunotherapy, and the relationship between the immune checkpoint PD-1/PD-L1 and the prognosis of various cancers is currently a hot topic in immunotherapy. High expression of PD-1 is more reflective of T cell exhaustion than intermediate or low expression [46]. Some studies have found that PD-1 is expressed on Tregs, and blocking PD-1/PD-L1 signaling can increase Treg proliferation [47]. Furthermore, these results showed that the expression of COL1A1 was significantly correlated with the regulation of multiple markers of T helper cells (Tfh, Th1, Th2 and Th17) in LGG. In brief, these results reveal the potential of COL1A1 to recruit and activate immune cells in brain lower grade glioma.

In summary, the high expression of COL1A1 is associated with poor prognosis of various cancers and higher levels of immune infiltration, especially in LGG. COL1A1 is likely to be a significant regulator of the immune infiltration in LGG patients and serves as a prognostic factor for LGG. Our research suggests that COL1A1 is a promising target for LGG research. Therefore, we strongly recommend further research on the biological impact of COL1A1 in this field.
